# Closing the loop: optimal stimulation of C*. elegans *neuronal network via adaptive control to exhibit full body movements

**DOI:** 10.1186/1471-2202-16-S1-O14

**Published:** 2015-12-18

**Authors:** Julia Santos, Eli Shlizerman

**Affiliations:** 1 Department of Applied Mathematics, University of Washington, Seattle, WA 98195, USA

## 

The *Caenorhabditis elegans (C. elegans) *worm is a well-studied biological organism model. The nervous system of C. elegans is particularly appealing to study, since it is a tractable fully functional neuronal network for which electro-physical connectivity map (connectome) is fully resolved [1,2]. In a recent work, we succeeded in establishing a computational dynamical model for the *C. elegans *nervous system and showed that robust oscillatory movements in motor neurons along the body can be invoked by constant current excitation of command sensory neurons (e.g. PLM neurons associated with forward crawling) and that their activation corresponds to low-dimensional Hopf bifurcation [3]. While these first results validated the model, it is exciting to learn how the nervous system transforms its oscillatory dynamics to the muscles to support robust full body movements (e.g. forward crawling) [4]. Moreover, using methods generically applicable to other neuronal circuits, it is intriguing to understand the optimal sensory stimulations that cause these movements to persist.

**Figure 1:**
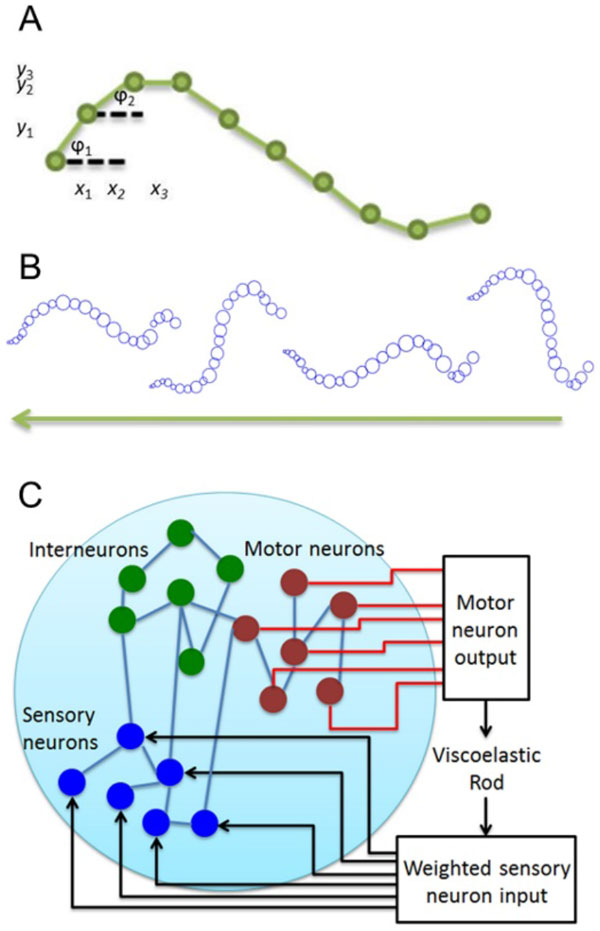
A: Structure of viscoelastic rod B: Viscoelastic rod-based simulation of C. elegans crawling during PLM excitation, videos available here C: Loop feeding transformed motor activity into sensory neurons.

We explore these questions by modeling the *C. elegans *musculature as a viscoelastic rod with discrete rigid segments [5], and map the neuronal dynamics such that they activate the muscles and deform the rod (Fig. 1A). When motor neuron activity stimulates muscles [2], this activation is translated into force applied to the rod, which moves in accordance with the physical properties of *C. elegans*. By stimulating the command PLM neurons, we establish for the first time that motor neuron dynamics are indeed producing coherent oscillatory full body movements that resemble forward crawling (Fig. 1B, videos available here: http://faculty.washington.edu/shlizee/celegans/).

We utilize our computational full body model to determine the appropriate sensory input for behavior, such as crawling, to persist after explicit external stimulation (touch) has ceased, as observed in experiments [5]. Since such persistence could be explained by a feedback loop between the environment and sensory neurons (Fig. 1C), we propose an adaptive control algorithm that extends existing recursive least squares-based algorithms (e.g. FORCE [6]). Our algorithm finds weights for synaptic input using a low-dimensional projection of motor neuron dynamics, and is capable of finding sensory input patterns that will lead to the desired movement.
